# Lab-in-the-field experiments: perspectives from research on gender

**DOI:** 10.1007/s42973-021-00088-6

**Published:** 2021-08-20

**Authors:** Lata Gangadharan, Tarun Jain, Pushkar Maitra, Joe Vecci

**Affiliations:** 1 Monash University, Melbourne, Australia; 2 Indian Institute of Management, Ahmedabad, India; 3 University of Gothenburg, Gothenburg, Sweden

**Keywords:** Lab-in-the-field experiments, Gender, C93, H41, J16

## Abstract

This paper highlights the contributions made by lab-in-the-field experiments, which are also known as artefactual, framed and extra-lab experiments. We present a curated sample of lab-in-the-field experiments and discuss how they can be conducted on their own or combined with conventional laboratory experiments, natural experiments, randomised control trials and surveys to provide unique insights into the behaviour of a diverse population. Using our recent research on gender and leadership, we demonstrate how lab-in-the-field experiments have offered new perspectives about gender differences in decision-making. Finally, we outline the ethical and implementational challenges researchers may face while conducting these experiments and share some of the strategies we employed to address them.

## Introduction

A defining feature of economics is that it investigates how the decisions of agents in society might change in the presence of competing incentives and diverse institutions. These decisions are often modelled using economic theory, yet assessing the empirical robustness of these theories is important for understanding how agents respond to economic and social stimuli in practice. Modern economic theory now recognizes that, in interactions where information asymmetries and expectations matter, agents act strategically subject to a range of factors, including cognitive limitations, preferences that contain elements of fairness, altruism and reciprocity, and social norms. Where these constraints were once lumped in the black box of “unobservables” (often categorized as “omitted variables” in econometric analyses), there is now a growing focus on improving our understanding of them, and how they affect agents’ behaviour (especially in ways that differ from what a traditional rational agent model would predict). While econometric and statistical tools allow us to test theoretical intuition using observational data, such naturally occurring data is often unable to accurately measure behavioural variables, and may not satisfy the assumptions required to provide clean causal inferences. Economic experiments are valuable tools to address these concerns.

Experiments can both help weed out incorrect theories and generate new causal insights that further economic theory as well as improve policy recommendations. In addition, where research questions are motivated by empirical regularities, such as the impact of individual (e.g., gender) and behavioural (e.g., confidence) characteristics on labour market outcomes, well-established evidence from experimental data can enable researchers to identify these regularities and further refine models of economic behaviour.

While early experiments in economics focused on testing specific aspects of economic theory (e.g., functioning of markets, predictions of game-theoretic models of behaviour, and individual choices), experiments are now used in a wide range of areas within economics including public economics, environmental economics, development economics and macroeconomics. The field also provides opportunities for interdisciplinary research due to its connections with psychology, political science, philosophy and sociology.

Researchers have designed both laboratory and field experiments in economics, with each having distinct advantages and limitations. In this paper, we explore the role played by field experiments in economics and focus on a particular kind of field experiment, referred to as a “lab-in-the-field” experiment. Our aim is not to offer a survey of the literature but to highlight some specific contributions of this method and provide select examples of the applications lab-in-the-field experiments can have in economics. Accordingly, we document topics that have benefited from the use of this method and discuss some of our recent research utilising lab-in-the-field experiments to understand gender differences in decision making and leadership. In the last section, we highlight the ethical and implementational challenges we encountered while planning and conducting these experiments, and share the strategies we employed to overcome these difficulties.

## What are field experiments?

Field experiments combine naturally occurring field data with aspects of controlled laboratory experiments, harnessing the benefits of randomisation in an environment that captures important features of the real world. This is particularly valuable in the social sciences since research subjects tend to have complex, heterogeneous behaviours, and sampling populations from different domains can permit stronger inferences (List & Reiley, 2008).

Economists have taken several approaches to classifying field experiments among other methodologies. Harrison and List (2004) identify six main distinguishing criteria: (1) the nature of the subject pool; (2) the nature of the information that the subjects bring to the task; (3) the nature of the commodity; (4) the nature of the stakes; (5) the nature of the task or trading rules applied; and (6) the nature of the environment that participants operate in. Using these criteria, they categorize experiments into four groups:

1. Conventional lab experiments: those that use a standard subject pool of students;
2. Artefactual field experiments: conventional lab experiments that use a “non-standard” subject pool
3. Framed field experiments: artefactual field experiments with “field context in either the commodity task, or information set that the subjects can use” (Harrison & List, 2004)
4. Natural field experiment: framed field experiments where the environment is one in which subjects naturally undertake the tasks being studied (such that participants do not know that they are in an experiment).

Charness et al. (2013) propose an alternative classification that comprise lab, field and extra-lab experiments. Under their approach, extra-lab experiments are similar to conventional lab experiments except for the venue and participant pool (which could include school students, online communities or villagers). Extra-lab experiments have similar characteristics to artefactual and framed field experiments in Harrison and List (2004). Samek (2019) provides a comprehensive summary of the advantages and disadvantages of field experiments. In recent years, researchers have also used the term lab-in-the-field experiments to refer to artefactual, framed and extra-lab experiments. This is the term we use in this paper.

**Fig. 1:**
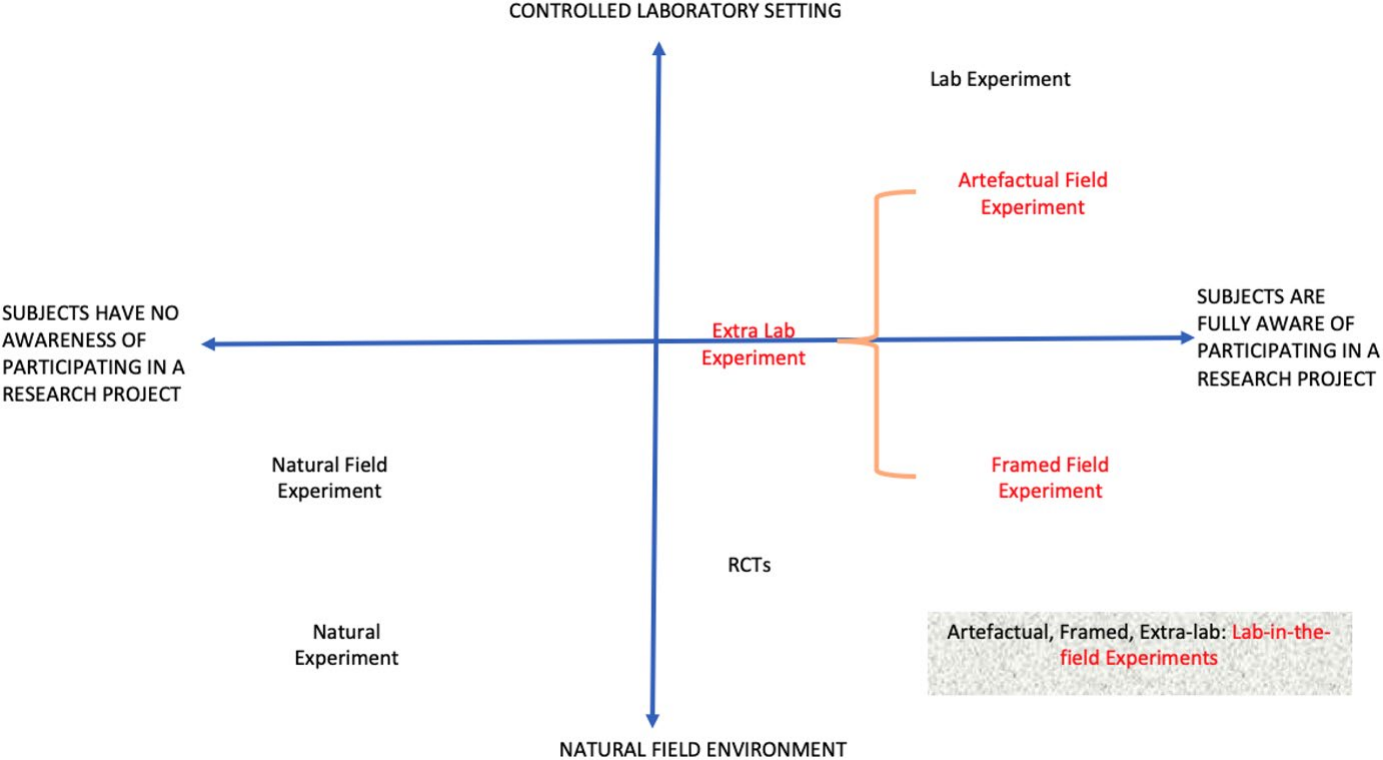
Classification of Experiments by Control and Awareness

**Fig. 2:**
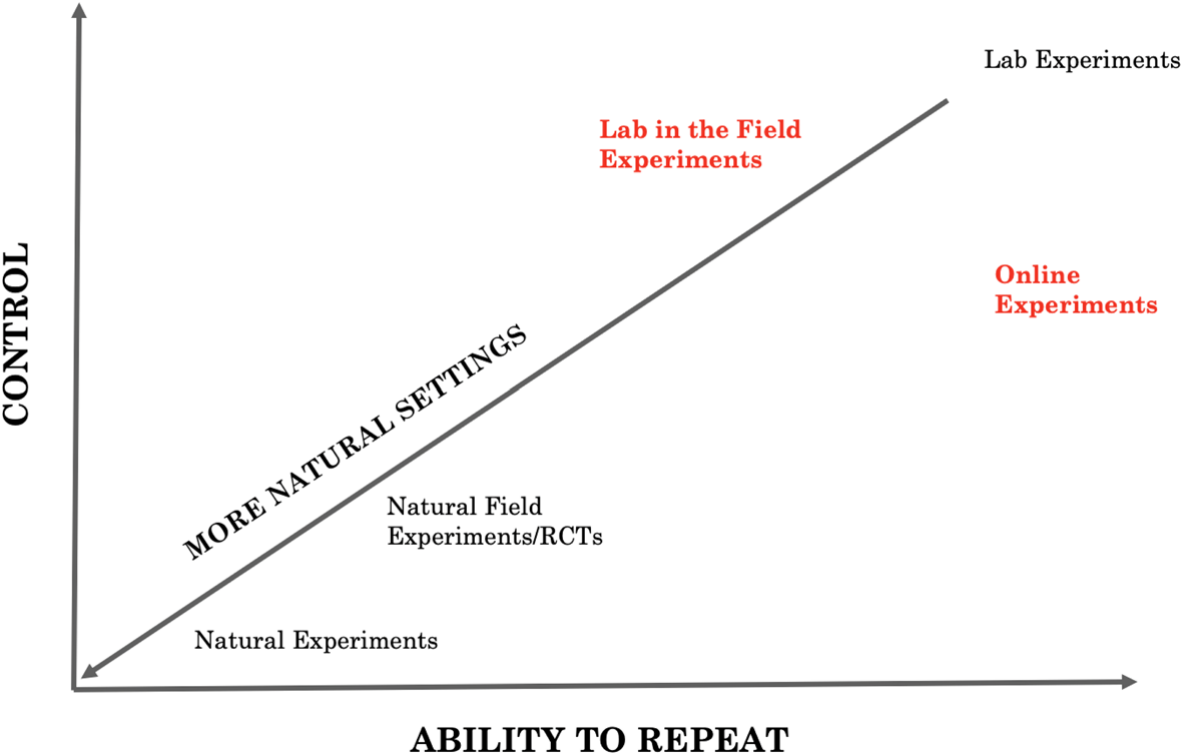
Classification of Experiments by Control and Ability to Repeat

Figures 1 and 2 present two alternative means of classifying experiments. Figure 1 uses the control that researchers have on the experimental environment, and how aware participants are about being in a research project, as the dimensions for classification. This figure refers to both natural field experiments and natural experiments. Though they share some features, in a natural field experiment, the researcher can often control the randomization, whereas in the natural experiment approach the researcher aims to find sources of variation in existing data that are “as good as randomly assigned” List and Rasul (2011). Randomised controlled trials (RCTs), which are also very popular in economics, share some characteristics with natural field experiments, however, a point of contrast is that RCT subjects are often aware that they are participating in a study as researchers elicit responses from them at various points throughout the research project.^1^ Figure 2 uses to control and ability to repeat the experiment as markers for classification. Figure 2 also includes online experiments, which have seen significant growth in recent years. Online experiments can be thought of as a form of lab-in-the-field experiment since they usually use non-student subjects as participants.

While these two-dimensional figures significantly simplify the nuances and characteristics of experimental research, they provide insight into the multi-dimensional choices and tradeoffs researchers face when choosing between methods. Which method is more preferable in a particular case depends primarily on the research question being investigated and the resources available to the researcher.

Overall, field experiments differ from conventional lab experiments along with a number of dimensions, including the subject pool, information that participants have, commodity, tasks, stakes and environment. Field experiments use “non-standard” subjects to the extent that they do not usually involve university students, the typical subject pool for conventional lab experiments. Instead, participants are drawn from the specific target population(s) in the economy to draw inferences from the unique characteristics, information or experiences they bring to the experiment and the decision-making process. In addition, field experiments often elicit choices over actual commodities (rather than abstract commodities, which is typically the case in lab experiments) to better account for whether the commodity itself affects behaviour (e.g. decisions about a real public good such as a road, as in, Beath et al. 2018).

Another point of contrast between lab and field experiments is that the latter provide context to suggest strategies and heuristics for undertaking the task at hand. Rather than viewing these as uncontrolled effects, the environmental cues and/or field experiences become central to determining behaviour and the nature of the task that the participants are being asked to perform. Accordingly, although the field context can reduce experimenters’ control, it also has the potential to increase the relevance and saliency of the task, allowing researchers to elicit more accurate responses from participants (depending on the research question). Both Harrison and List (2004) and Charness et al. (2013) recommend that field approaches be considered methodologically complementary to lab experiments.

By attempting to simulate “real life” as closely as possible, field experiments can also enhance the external validity, or generalizability, of experimental findings. In this way, field experiments can offer a distinctive and new source of empirical evidence, which can then be compared, contrasted, reconciled and eventually intertwined with evidence from non-experimental and lab methods.^2^

## Lab-in-the-field experiments

The focus of this paper is on a specific type of field experiment referred to as a lab-in-the-field experiment, also known as an artefactual, framed or extra-laboratory experiment. Viceisza (2015) provides a survey of the literature on lab-in-the-field experiments.

In economics, lab-in-the-field experiments have been used: (1) to examine the decisions of a broader range of subjects, extending our understanding of human behaviour beyond a few select communities in the western world (Heinrich et al., 2010); (2) to compare decisions across subject pools; and (3) in combination with other empirical approaches, such as conventional lab experiments, natural experiments (comprising both natural shocks and policies), randomized control trials and observational data. In this section, we provide examples of each case to demonstrate the contributions made by this experimental approach.

*Lab-in-the-field experiments to examine subject pool differences*: Since lab-in-the-field experiments employ participants from specific contexts, they can help examine the behaviour of unique populations and determine whether this behaviour is consistent across subject pools. Reflecting the breadth of possible subjects, researchers have conducted experiments with public servants (Alatas et al., 2009), nurses (Cadsby & Maynes, 1998; Barr et al., 2009; Hanna & Wang, 2017); CEOs (Fehr & List, 2010; List & Mason, 2011), managers in Chinese state-owned enterprises (Cooper et al., 1999), married couples (Dasgupta & Mani, 2015; Iversen et al., 2011; Masekesa & Munro, 2020), prisoners (Cameron et al., 2019; Guo et al., 2020) and politicians (Banerjee et al., 2020; Chaudhuri et al., 2020, 2021).^3^

The study by Alatas et al. (2009) demonstrates the usefulness of using non-standard subjects. It finds that while both Indonesian public servant participants and Indonesian student subjects are corrupt, public servants are significantly *less* likely to engage in corruption than student subjects. Using public servants as participants allowed the researchers to tease out the mechanisms underlying their results, revealing that experience (rather than selection into public service) is the main driver of the behaviour. Similarly, Hanna and Wang (2017) used a sample of students and government workers (nurses) to find that dishonest individuals within both groups prefer to enter government service. More recently, Chaudhuri et al*.* (2020) and Chaudhuri et al. (2021) compare the behaviour of politicians and ordinary citizens using survey and experimental data covering village councils in rural India. They find that inexperienced (and first-time elected) village council politicians are less dishonest and more pro-social than ordinary citizens. However, this idealism appears to wear off over time.

*Lab-in-the-field experiments as a complement to traditional laboratory experiments*: The lab-in-the-field experiment can be used *ex post* to test for external validity. For example, Blackburn et al. (1994) estimated a statistical model of subject response using two different convenience samples: college students, and field subjects drawn from a broad range of churches in the same urban area. The church sample exhibited a much wider variability in socio-demographic characteristics, with ages ranging from 21 to 79 years compared to 19–27 years for the student subject pool. They found that predicting the behaviour of students based on the church-estimated behavioural model was extremely accurate, but predicting church behaviour from the student-estimated behavioural model led to wide forecast variances. It follows that, by offering access to a broader range of research subjects, lab-in-the-field experiments can improve predictions of the behaviour of the target population.

*Lab-in-the-field experiments to study responses to natural shocks*: Natural occurrences and disasters have often been used by researchers to understand decision-making as the disaster can be considered an exogenous shock. Lab-in-the-field experiments have been conducted in areas which were exposed (more/less) to a disaster to exploit this plausibly exogenous variation. For example, evidence from developed countries is provided by Eckel et al. (2009), Page et al. (2014) and Hanaoka et al. (2018), while evidence from developing countries is supplied in Cameron and Shah (2015), Cassar et al. (2017), Brown et al. (2018) and Islam et al*.* (2020). Maitra and Neelim (2021) summarize some of the recent studies on this topic. The COVID-19 pandemic is an important example of a natural shock the world is currently experiencing. Campos-Mercade et al. (2021) examine individual responses to this pandemic and find that pro-sociality predicts health behaviour during the pandemic.

In addition, lab-in-the-field experiments have been used to examine the long-term effects of exposure to violent conflicts, wars and resource scarcity. For example, Prediger et al. (2014), Cecchi and Duchoslav (2018) and Gangadharan et al*.* (2020) find that resource scarcity and exposure to wars (including growing up during wars) can have long term behavioural effects, especially on antisocial behaviour.

*Lab-in-the-field experiments to better understand the effect of policies and programs*: Researchers have also employed lab-in-the-field experiments in settings where important new policies with overarching impacts have been initiated by governments. Economists have used the exogenous shift in policies to provide useful insights into how a policy change influences savings, consumption patterns, education and labor market outcomes (mainly using observational data). However, measuring and examining behavioural patterns using such data is difficult. In this situation, lab-in-the-field experiments can be leveraged to explore these patterns in a robust way. For example, Cameron et al. (2013) use the one-child policy in China as a backdrop to examine the social, risk and competitive preferences of those who were single children as a consequence of this policy, while Gangadharan et al. (2016), and Gangadharan et al. (2019) (discussed in more detail in Sect. 4) use the randomised affirmative action policy in India to investigate gender differences in leadership in a public goods experiment.

One concern when investigating the effect of policy changes that cover large populations, and therefore use large data sets, is that data collection is not targeted to address specific questions. Even where conducting a sophisticated data collection exercise specifically targeted to the program is possible *ex post*, pre-program data is generally difficult to obtain. Policymakers have found lab-in-the-field experiments to be particularly useful in such cases. For example, Ludwig et al. (2011) emphasize the importance of uncovering the mechanism through which treatment effects occur in complex policy environments; an insight that in many cases can be derived from a relatively simple set of experimental treatments, “especially when the extra-lab (lab in the field) result is benchmarked against its lab precursor” (Charness et al., 2013). By conducting lab-in-the-field experiments directly on a target population or at the location of a potential policy intervention, policy makers can examine its effect on a small scale before fully implementing a project with potentially large consequences. Plott (1982) and Smith (1994) mention this approach as being similar to a testbed, which allows for rigorous, transparent and replicable testing of new scientific methods and ideas. Such testbeds have been widely used to develop and fine-tune policies (e.g. in pollution markets and spectrum auctions) in many parts of the world.

*Lab-in-the-field experiments combined with Randomised Controlled Trials*: RCTs are explicitly designed with a research purpose in mind, with participants randomly assigned to treatment and control arms. RCTs measure outcomes that can accurately be captured using survey data. For instance, an RCT that varies access to cash transfers will generally measure changes in income, consumption and poverty through baseline and end-line surveys. However, as discussed by Falk and Heckman (2009) and Barrett and Carter (2010) among others, many RCTs fail to measure and illuminate the mechanisms behind any variation in outcomes, especially behavioural mechanisms. In contrast, lab-in-the-field experiments are specifically designed to understand and measure behavioural mechanisms.^4^ For example, Maitra and Mani (2017) use a lab-in-the-field experiment to examine the role played by risk attitudes and preferences for competitiveness in understanding the causal (treatment) effects obtained from a labor market training program.

Lab-in-the-field experiments are particularly useful where behavioural mechanisms are relevant as they are generally less costly than RCTs and allow for multiple observations of the same individual under varying but controlled conditions. For instance, subjects can participate in a public goods task across multiple periods, with a different partner assigned each period. In addition, lab-in-the-field experiments can test very specific theoretical hypotheses, which is difficult with RCTs because controlling variation in the decision environment in a naturally occurring setting is often not feasible.

As such, lab-in-the-field experiments are useful complements to a range of field experiments, from those arising to examine the impact of disasters or the evaluation of government policies and programs (referred to as natural experiments in Figs. 1 and 2) to RCTs examining the effectiveness of the treatment imposed by researchers or policymakers. Despite a somewhat artificial setting, they offer greater control over the environment which allows for a better understanding of the causal mechanisms. This suggests conducting both studies in tandem will improve interpretation as well as the replicability of findings.

*Lab-in-the-field experiments as a complement to surveys*: In cases where responses to survey questions are prone to social desirability bias, lab-in-the-field experiments can provide a more accurate instrument compared with field surveys. For topics such as discrimination, corruption, dishonesty, fairness and redistribution, direct questions can lead to biased results because the respondent may be more inclined to provide an answer that is socially acceptable rather than reflective of their true attitudes or preferences. For example, on a survey investigating attitudes towards daughters working outside the home, parents may report approving of such behaviour only because this response is socially acceptable. They may also do this to project a favorable image to the surveyor or themselves, or to avoid receiving negative evaluations. While a researcher could test and reduce social desirability bias using methods such as list experiments (Blair & Imai, 2012), bounding demand effects (de Quidt et al., 2018)) or testing sensitivity to report socially desirable responses (Dhar et al., 2020), an alternative is to conduct a lab-in-the-field experiment. In experiments, participants may not know the true aim of the design, which reduces the probability that subjects will try to behave in a socially desirable manner.

The fact that, in lab-in-the-field experiments, financial incentives are associated with the choices made by participants within the experiment means that participants are more likely to reveal their true preferences. This is important because monetary payments help link choices to behaviour outside the experimental setting (as choices in the real world often involve payoff consequences) and encourage subjects to take their decisions seriously (Cardenas, 2000; Cardenas & Ostrom, 2004; Smith, 1989); behaviour in the laboratory becomes reliable and “real” when subjects take decisions with meaningful economic consequences because they perceive their own behaviour as relevant and experience real emotions.^5^ Researchers have experimentally tested the effect of incentives in experimental economics and have shown that in many (but not all) tasks, subjects exert more effort if monetary earnings are tied to performance. For example, Camerer and Hogarth (1999), Gneezy and Rustichini (2000) and Erkal et al. (2018) provide lab and field evidence on the impact of monetary incentives. Relatedly, researchers have shown that stake size matters. For instance, using a lab-in-the-field experiment, Leibbrandt et al. (2018) show that participants are more likely to lie when the stakes are very large. Related to this, lab-in-the-field experiments enable researchers to understand and measure behavioural characteristics that have traditionally been considered unobservable in surveys (e.g. preferences, beliefs and norms) as either the dependent or independent variable “at a refined level that is unlikely to be feasible by other empirical methods” (Viceisza, 2015). In the subsection below, we outline some of these characteristics.

For the reasons mentioned above, researchers are increasing combining survey and lab-in-the-field methods. For instance, Bartling et al. (2009) report results from several economic experiments embedded in a household survey study within the German Socio-Economic Panel, a large representative survey of private households in Germany. Sarsons et al. (2021) similarly use administrative data on publications in economics journals to determine gender differences in evaluations and complement this with an online lab in the field experiments to examine the mechanisms underlying their results. Bhalotra et al*.* (2021) also combine surveys and lab in the field experiments to understand responses to the religion of a group leader.

### Lab-in-the-field experiments: some commonly used games/tasks

Lab-in-the-field experiments have many applications, and most tasks conducted in a conventional lab have the potential to be implemented in the field with some modification. In this section, we provide a brief outline of the most common lab-in-the-field tasks. These include individual choice experiments (such as those conducted to elicit risk and time preferences), experiments to elicit other-regarding preferences and experiments to elicit beliefs about behaviour and social norms.

*Risk and Time Preferences*: A large body of research shows that individual risk preferences play an important role in decision-making across many domains. For example, risk preferences have significant effects on occupational choices, schooling decisions, technology adoption, financial decisions and the choice to be enrolled in skills training programs (see Castillo et al., 2010; Dercon & Christiaensen, 2011; Belzil and Leonardi, 2009; Liu, 2013; Dasgupta et al., 2015). Maitra and Neelim (2021) provide a brief survey of this literature. In recent work, the risk preferences of individuals have also been shown to be relatively stable in developed countries but highly unstable in developing countries (Cardak et al., 2021). Risk preferences are often elicited with the use of a lottery. Depending on the elicitation method, subjects can be classified as risk averse, risk neutral or risk takers. Laboratory experiments on risk (such as Eckel & Grossman, 2008; Gneezy & Potters, 1997; Holt & Laury, 2002) were inspired by the early studies of Binswanger (1980), who conducted risk elicitation experiments with farmers in India.

Time preference, which describes the amount someone values the future relative to the present, is another fundamental factor for explaining decisions. It has been shown that impatient individuals may be reluctant to save or to invest in their future (see Golsteyn et al., 2014; Newell & Siikamäki, 2015), while more patient women may have a greater incentive to educate themselves and their children, since education may be understood as a long-term investment in the future (Duflo, 2012). Individuals may also exhibit naiveté, such that they underestimate the degree of future present bias. Naiveté may lead individuals to delay high return investments that have a short-run utility cost (even if small) because they incorrectly anticipate making these investments later.

Time preferences are typically elicited by asking participants to choose between a smaller-sooner option that gives them a relatively small financial reward relatively soon and a larger-later option that gives them a larger reward after a longer delay.^6^ These experiments are often conducted in conjunction with risk experiments. For example, Tanaka et al. (2010) conduct incentivized time and risk preference experiments in rural Vietnam and find that in villages with higher mean income, people are less loss-averse and more patient. This shows that household income is correlated with patience but not with risk.

*Pro-social preferences*: Measuring pro-social preferences using field experiments is common. Tasks to elicit pro-social preferences include dictator games as a measure of altruism, public goods games to measure cooperation, trust games to measure trust and trustworthiness, and ultimatum games to measure fairness in bargaining. Social preference tasks have also been used to investigate cross-country differences in behaviour and subject pool (Henrich, 2000 and Henrich et al., 2001), in-group and out-group biases across societies (Afridi et al., 2015; Chen & Li, 2009; Gupta et al., 2018), patterns of behaviour across matrilineal and patriarchal societies (Andersen et al., 2013; Banerjee et al., 2015a, 2015b; Gangadharan et al., 2021; Gneezy et al., 2009; Mukherjee, 2018), and patterns of evolution by examining the behaviour of school children and adolescents (Harbaugh & Krause, 2000; Sutter et al., 2013).

*Anti-social preferences*: Researchers have also examined anti-social behaviour using lab-in-the-field experiments (e.g. Prediger et al. (2014) and Gangadharan et al*.* (2020), explore anti-social preferences of pastoralists in Namibia facing resource scarcity and individuals exposed to the Cambodian genocide, respectively). A voluminous literature uses the die-tossing task (Fischbacher & Föllmi-Heusi, 2013) or variants of it (where participant earnings depend on self-reported outcomes) as a reliable measure of dishonesty and corruption at both the individual and macroeconomic level. Several studies have used such tasks to examine dishonesty amongst different groups of the population. These include bankers (Cohn et al. (2014)), prisoners (Cohn et al. (2015)), public and private sector aspirants (Banerjee et al., (2015a, 2015b)), civil servants and nurses (Hanna and Wang (2017)), students and teachers (Cohn and Maréchal (2019)), milk vendors (Kröll and Rustagi (2020)) and elected politicians (Chaudhuri et al*.,* 2020, 2021). Gächter and Schulz (2016) and Olsen et al. (2019) have conducted cross-sectional studies to examine how behaviour in the die-tossing task is correlated with country-level measures of corruption such as the Corruptions Perceptions Index. They find that citizens of more (less) corrupt countries tend to be less (more) truthful in reporting their outcomes in the die-tossing task.

*Eliciting beliefs*: Since beliefs can have a large influence on decisions, eliciting beliefs about behaviour within an experiment is increasingly common. For example, whether an individual contributes to a public good is in part influenced by whether they believe others will also contribute. While a useful summary of elicitation methods can be found in Schotter and Trevino (2014) and Schlag et al. (2015), the core idea is to elicit from participants what they believe occurred (e.g. “how much did you think your group members would contribute in the public goods game?”) with financial incentives based on how accurate a participant’s belief is with respect to the actual behaviour. Bursztyn et al. (2020) report an interesting application in relation to beliefs by studying women working in the labour force in Saudi Arabia.

*Social norms*: While the inclusion of social norms within economic models is relatively new, research on social norms has produced significant evidence revealing their importance in understanding behavioural mechanisms (Gangadharan et al., 2016; Dimant et al., 2020; Jayachandran, 2020). A common method to measure social norms is to use a coordination game (Krupka and Weber, (2013)), where participants are asked to guess what they think others believe is socially appropriate in a given context. Responses are often given on a four-point scale ranging from very socially appropriate to very socially inappropriate. Unlike standard belief questions, these questions specifically focus on the social appropriateness of behaviour (for instance, respondents could be asked whether it is socially appropriate for a male to work as a homemaker). This method is useful because subjects have an incentive to reveal what they think others believe is socially appropriate, not what they believe themselves (Exley et al., 2020; Gächter et al., 2013). Varying the reference group is possible, which allows researchers to elicit what respondents think females believe as compared to what males believe. We use this method in the research described in Sect. 4.

## Does the gender of the decision-maker matter?

### Gender and leadership: Impact of affirmative action

In this section, we describe lab-in-the-field research focusing on gender and leadership, especially that reported in Gangadharan et al. (2016) and Gangadharan et al. (2019), which illustrate how lab-in-the-field experiments can be combined with a natural policy change to provide insights into behaviours that can undermine the leadership of women.

Research in this area has grown rapidly as a response to the increasing concern about the poor representation of women in leadership positions. Women constitute only 7% of all heads of government, 4.8% of Fortune 500 company CEOs, 7% of central bank governors and 2.5% of self-made billionaires. When given the opportunity, women leaders often make different policy decisions compared to men. This has been shown in terms of workforce reductions (Adams & Ferreira, 2009; Ahern & Dittmar, 2012; Matsa & Miller, 2013; Eckbo et al*.* 2019; Bertrand et al., 2019), spending on vulnerable populations (Chattopadhyay & Duflo, 2004a, 2004b; Edlund & Pande, 2001; Lott & Kenny, 1999; Pande & Ford, 2012) and corruption (Brollo & Troiano, 2016). Together, this suggests that more women in leadership positions could offer substantial economic and social benefits. A prominent approach to increasing the number of women in leadership positions has been to use affirmative action policies, such as quotas or reservation of seats for women in leadership positions.

Observational data from developed countries on the effect of gender quotas on female political engagement provides mixed evidence. In support of this measure, O'Brien and Rickne (2016) find that a quota for women introduced in Sweden increased the number of women perceived as qualified for higher positions and accelerated women's representation in leadership positions, while De Paola et al. (2010) and Baltrunaite et al*.* (2014) show that even temporary quotas increased women's representation in politics and led to higher quality politicians (both men and women) being elected to office. Conversely, Bagues and Campa (2020) and Lassebie (2020) find no evidence that quotas in Spain and France led to systematic improvements in the career progression of women.

Ultimately, the likelihood of women becoming successful leaders depends on whether citizens (both men and women) accept them in leadership positions. However, little is known about how men and women respond to female leaders. The behavioral response towards and of leaders can be a difficult question to answer using observational data. We thus investigate this using two lab-in-the-field experiments, which present some unique advantages in this context.^7^ First, the allocation of leader roles in the experiments enables data to be collected from female decisions makers, which is often not feasible using observational data due to the limited number of women in leadership roles. Second, the randomized assignment of leadership status avoids selection issues relating to the leader’s identity, meaning that the citizens’ actions in response to the leader’s gender can be interpreted as causal. This is difficult using observational data, as other leader characteristics can confound how the leaders are perceived. Third, with observational data there is no straightforward way of disentangling the preferences and reactions of the leaders and the citizens. However, experiments can measure the decisions of both leaders and citizens and separately attribute their decisions to the treatment they are in. Fourth, the experimental approach reduces social desirability bias (as noted in Sect. 3). Finally, the field context allows the experimental findings to be placed in the context of participants’ actual exposure to male and female leaders in their villages.

The first experiment is a *leadership experiment* conducted in 40 villages, with 956 participants, in three districts in the state of Bihar in India. The design utilises changes introduced by the 73rd Amendment to the Indian Constitution, ratified by the National Parliament in April 1993, which codified a system of rural local governance (called the “Panchayati Raj”) and also introduced quotas for women in every level of rural local governance. In each state, at least one third of all seats in every village, sub-district or district level council were to be reserved for women, and across the state at least one third of all head of village council positions were to be reserved for women.^8^ Bihar introduced the Panchayati Raj system later than many other states, and is categorized as a late adopter of the 73rd Amendment to the Indian constitution; the first Panchayati Raj election in Bihar was held in 2001. However, Bihar was the first state to increase the proportion of seats to be reserved for women to 50%, a norm later adopted by most states. In 2014, when these experimental sessions were run, Bihar had participated in three Panchayati Raj (including village council) elections: in 2001, 2006 and 2011.

We use this policy, and the results from the elections, to define female-headed and male-headed villages. We classify a village as a female-headed village if it had at least one female head in the three village council elections, and a male-headed village if it never had a female head. Those living in male-headed villages have very limited experience with women as leaders.^9^

The leadership experiment is a modified one-shot linear public goods game. Participants are randomly assigned to groups. Each group consists of two men and two women. Group composition is public information. One group member is randomly and anonymously chosen as the group leader; the remaining three group members can be thought of as citizens. There are multiple groups in each session. Participants are not provided any information about the identity of the other members of the group, apart from the gender composition of the other group members. Notably, half the groups in each session have female leaders. As in a standard public goods game, each participant receives an endowment (in this case, Rs. 200). The game has two stages. In the first stage, the leader proposes a non-binding contribution (${p}_{i}$) towards the group account. The leader’s proposal is communicated anonymously to group members. In the second stage, all group members, including the leader, simultaneously contribute towards the group account, with payoffs calculated as $\Pi ={c}_{i}+\frac{\alpha {\sum }_{i}g\_i}{4}; \, {c}_{i}+{g}_{i}=200; \, \alpha =2.$ Here, ${g}_{i}$ is the amount that individual members contribute to the public account, and ${c}_{i}$ is what is left for their private account. The leader’s proposal is not binding (cheap talk) so in the second stage they are not restricted to contributing what they had proposed in the first stage of the experiment i.e., ${p}_{i}>=<{g}_{i}$.

We conduct two treatments with an approximately equal number of participants. *Gender revealed* where the group members are informed of the leader’s proposed amount and the leader’s gender and *Gender not revealed*, where the group members are only informed about the leader’s proposed amount. The treatments allow us to observe the behaviour of male and female members towards male and female leaders. Further, we can exogenously vary the observability of the leader’s identity, allowing us to understand how citizens will behave when the leaders gender is unknown.^10^ Both these aspects are challenging to implement using other empirical approaches.

We, therefore, have two sources of random variation: (1) treatment variation arising from the experimental design (variation in the information about the gender of the head of the group, i.e., male head, female head or the head of the group is not known); and (2) exogenous variation in exposure to female heads of village councils, introduced through the affirmative action policy.^11^

**Fig. 3:**
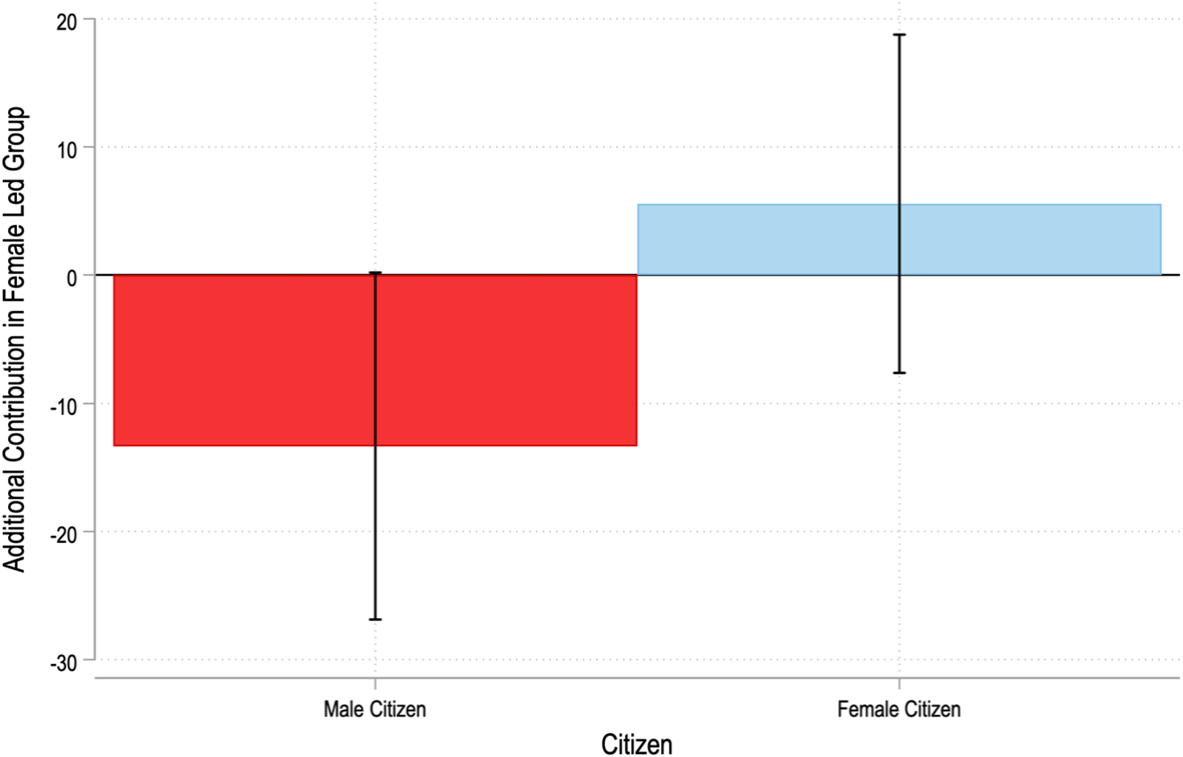
Additional Contribution in Female Led Groups, by Male and Female Citizens. The bars denote the additional contribution in female-led groups by male and female citizens (a negative amount is shown if the contribution is higher in male-led groups). The capped lines denote the 90% confidence intervals. For further details, see Table 4 in Gangadharan et al. (2016)

Figure 3 depicts the key difference estimates reflecting the additional contribution made by male citizens and female citizens in female-led groups (relative to male-led groups), computed using a multivariate regression analysis. We find that male citizens contribute Rs. 13 (or 7% of their endowment) *less* in female-led groups relative to male-led groups, while female citizens’ contributions show little variation based on the leader’s gender. We refer to this as male *backlash* against female group leaders.

**Fig. 4:**
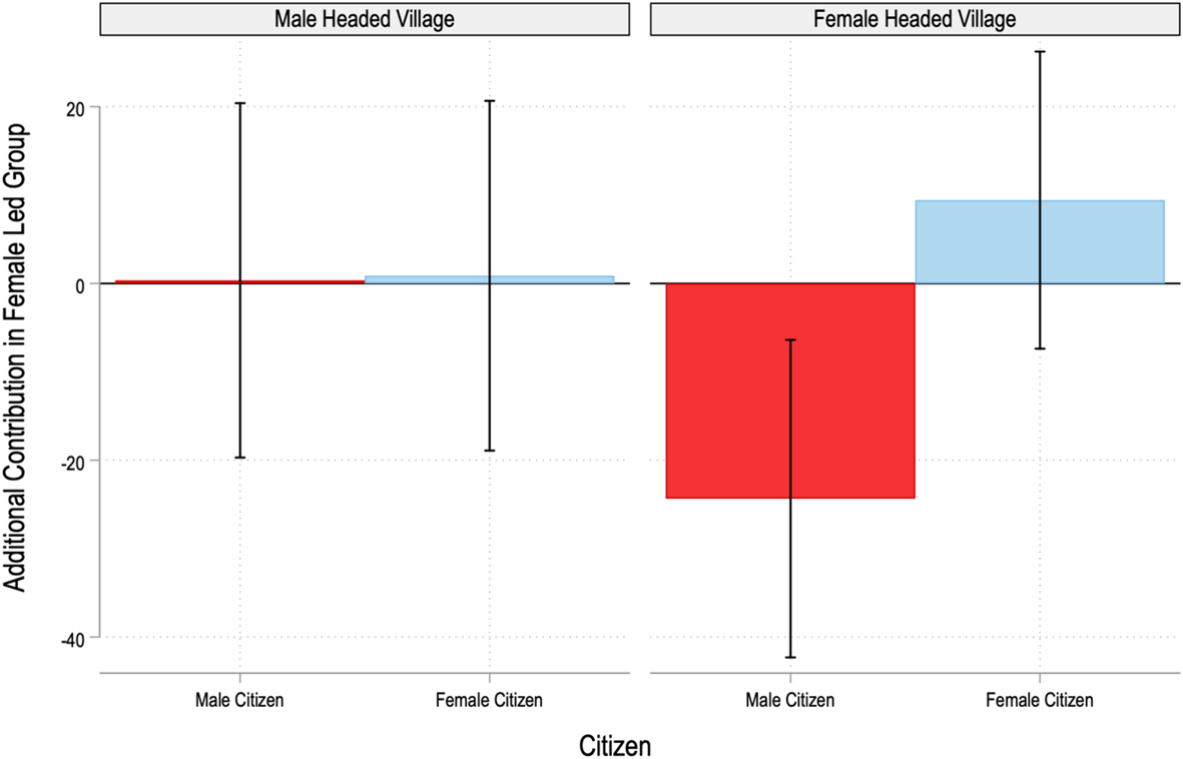
Additional Contribution in Female Led Groups by Male and Female Citizens in Male and Female-Headed Villages. The bars denote the additional contribution in female-led groups by male and female citizens in male and female-headed villages (a negative amount is shown if the contribution is higher in male-led groups). The capped lines denote the 90% confidence intervals. For further details, see Gangadharan et al. (2016)

To understand whether these differences differ by the gender of the head of the village council, we compare behaviour in female-led groups in both male and female-headed villages. Figure 4 presents the difference estimates diagrammatically. We find that male citizens contribute significantly less to female led groups (relative to male led groups), but only in female-headed villages. The estimated effects imply that men contribute Rs. 24 (or 12% of their endowment) less to female-led groups in female-headed villages. Irrespective of whether the village is male headed or female headed, female citizens do not contribute differentially in male and female-led groups. This reveals that the male backlash against female leaders is driven by a male response against female leaders in female-headed villages.

To better understand the mechanisms driving our key results (Figs. 3 and 4), we conducted a second experiment. This was a *social norms* experiment. It was conducted in 21 villages with 267 individuals. The participants for the social norms experiment lived in the same districts (but not in the same villages) as those who participated in the leadership experiment. Participants undertook three tasks that described possible decisions made by participants in the original leadership experiment as well as a number of vignettes.^12^ Participants in the social norms experiment rated each decision along a four-point Likert scale (very socially inappropriate, somewhat socially inappropriate, somewhat socially appropriate and very socially appropriate). Incentivization encouraged participants to reveal their beliefs about others’ actions rather than their own preferences. The participants’ aim was to match their responses with those of others (similar to a coordination game). As discussed in Sect. 3.1, the use of incentivization distinguishes this experiment from a survey measure that elicits norms, and increases the cost of responding in a socially desirable manner.

To understand the motivations for the male backlash observed in the leadership experiment, we focus on responses in one specific task in the social norms experiment, namely that asking subjects to rate the social appropriateness of a male/female citizen contributing Rs. 0, 50, 100, 150 or 200 to the group account, when the group leader is male or female. We find that males believe that it is more socially appropriate for men to contribute 50% or less of their endowment to a female-led group (i.e., it is socially less costly for males to contribute less to female-led groups). This pattern of expectations about the behaviour of male and female citizens is consistent with the observed backlash against female leaders.

However, this is not the only explanation for such a backlash. A second potential driver is the identity crisis that men may face when women are appointed to leadership positions. Males may view leadership and power as the domain of men, and may experience a loss of identity when women encroach upon this domain. Gender is a particularly strong aspect of identity, and the appointment of women in a leadership position may lead to a violation of male identity. Individuals (in this case, men) who believe their identity is being violated might act out to bolster a sense of self or to salvage a diminished self-image. Consistent with this notion, male identity is more conspicuous in female-headed villages, which is where men’s role is being challenged. As Fig. 4 documents, backlash against female leaders is being driven by the behaviour of men in female-headed villages.

What about the leaders themselves? We argue that the social environment is another potential barrier to the effectiveness of women leaders. This is because women leaders may respond negatively to the expectation that men are likely to react differently to them (especially those in authority due to an affirmative action policy). Such a response could reduce trust and cooperation, and consequently decrease social and economic welfare in the long run, further diluting the effectiveness of women’s leadership.

**Fig. 5:**
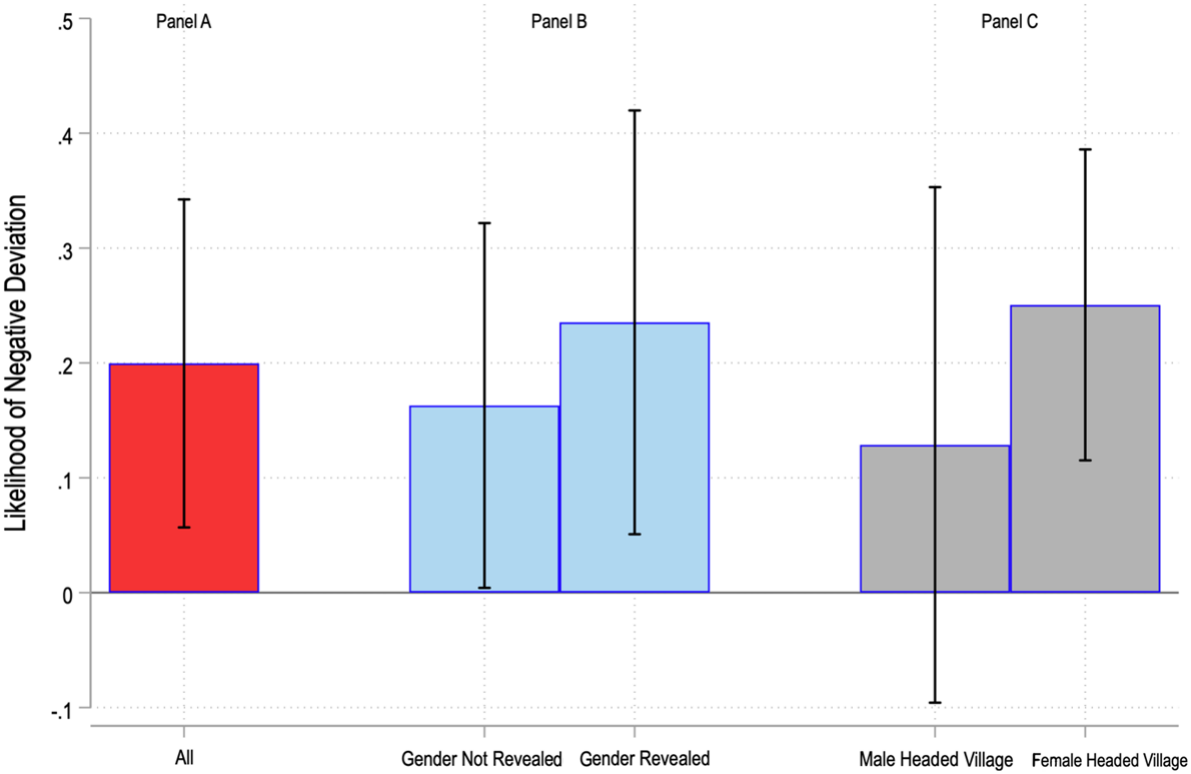
Gender Differences in the likelihood of Negative Deviation. The bars denote the additional likelihood of negative deviation (${p}_{i}-{g}_{i}$) by women leaders (positive if women deviate more and negative if men deviate more). The capped lines denote the 90% confidence intervals. For further details, see Gangadharan et al. (2019)

Accordingly, we consider whether there is a gender difference in leaders’ propensity to contribute less to the public good than stated in their proposals. The results presented in Fig. 5, Panel A show that female leaders are, on average, 20 percentage points more likely to choose to deviate negatively from their stated proposals compared to male leaders (i.e., ${g}_{i}<{p}_{i}$). However, a related and potentially more important question is whether these differences in behaviour reflect differences in male and female leaders’ inherent preferences, or whether they reflect differential responses by male and female leaders to the social environment. This is important because policy prescriptions may differ depending on the cause of the behaviour. To examine whether the social environment is driving the results, we first consider deviations by leaders depending on whether the gender of the group leader is revealed to the citizens (the treatment effect). The results are presented in Fig. 5, Panel B. They indicate that female group leaders are 23.5 percentage points more likely to deviate negatively in gender revealed sessions, and only 16.3 percentage points more in gender not revealed sessions.

Second, we consider the influence of the gender of the village head. The likelihood of negative deviation in female and male-headed villages is presented in Fig. 5, Panel C. Compared to male leaders, female leaders are significantly more likely to exhibit negative deviation in female-headed villages. The likelihood of observing a negative deviation is not statistically different for male and female leaders in male-headed villages.

Taken together, these results suggest that, when the social environment is such that women anticipate men in their group will not cooperate (either because the men know the gender of the group leader or because they are in female-headed villages), they react negatively by reducing their contributions as compared to their proposals.

### Further explorations of the social environment

One way in which researchers have examined the impact of the social environment on gender is to conduct lab-in-the-field experiments in locations with different cultural and historical gender-related institutions. An example of this is Gneezy et al. (2009)’s utilisation of lab-in-the-field experiments to explore the underpinnings of gender differences in competitive attitudes across the Maasai in Tanzania (a patriarchal society) and the Khasi in India (a matrilineal society). They find that, among the Maasai, men opt to compete at roughly twice the rate of women, but among the Khasi this phenomenon is reversed. This finding suggests that nurture may be a stronger determinant of competitive inclination than nature. Andersen et al. (2013) extend this line of argument by investigating whether women are born less competitive than men, or whether they become so through the process of socialization, by comparing the competitiveness of children in matrilineal and patriarchal societies. They find no gender differences at any age in the matrilineal society, but that girls become less competitive around puberty in the patriarchal society. Researchers have also investigated gender differences in negotiation (Andersen et al., 2018), public good provision (Andersen et al., 2008; Banerjee et al., 2015a, 2015b), investment in risky microfinance projects (Mukherjee, 2018), and dictator game giving (Gong et al., 2015) in matrilineal and patriarchal societies.

Building on this body of research, we aim to better understand the role of the social environment in the process of decision-making. As reported in the previous section, we found that male and female leaders are treated differently by group members. In Gangadharan et al. (2021), we aim to further explore this issue by studying whether the choices of male and female decision-makers are evaluated differently by independent third parties (i.e. participants who, unlike a group member, are not monetarily affected by the actions of the decision-maker). As Fehr and Fischbacher (2004) argue, unaffected third parties, by their willingness to incur a cost to sanction decision-makers for the transgression of norms, reveal the true normative standards of behaviour. Such third-party evaluations are universal across cultures and across time (see Greif, 1993, 1994; Henrich et al., 2006).

The key research questions we examine are: (i) whether male and female third parties evaluate the actions of male and female decision-makers differently; and (ii) whether these evaluations vary across social environments. We conduct two lab-in-the-field experiments. Both involve a variant of a third-party dictator game (Fehr & Fischbacher, 2004), consisting of two stages. In Stage 1, which is similar to a standard dictator game, a decision-maker (a “Proposer”) chooses how to divide an endowment between themselves and a passive recipient. In Stage 2, a third party (the “Judge”) evaluates the choice and can choose to punish the Proposer for their actions. Punishment is costly, but by investing $\pi$ the Judge can increase or decrease the Proposer’s income by $3\pi$. If the salient norm is one of equal distribution, theories of social preference would suggest that Judges care about the norm of equality and may choose to punish transgressions of this norm. We investigate whether there are gender differences in punishments and whether these are influenced by the social environment.

The variation in the social environment is captured by conducting the two experiments in regions of India where norms relating to gender differ widely. Experiment 1 is conducted with matrilineal societies in the state of Meghalaya. Experiment 2 is conducted in the state of Haryana, which is characterised by strong patriarchy, regressive gender norms and strong gender bias against women. We find that the social environment can be critical in how men and women are evaluated for transgressing norms. In a more gender-equal environment (Experiment 1), both male and female decision-makers are punished. However, men are more likely to punish male decision-makers, while women are more likely to sanction female decision-makers, for transgressions. In an environment with significant gender biases (Experiment 2), female decision-makers are significantly more likely to be punished than men for the same transgression. In this case, however, male evaluators are more likely to punish female transgressions. We infer that male-dominated environments such as that in Experiment 2 can be more susceptible to stereotypes (e.g. that women are less selfish than men) so, for the same transgression, women are punished more heavily when they act against the stereotype.

These results suggest that the social environment is crucial for understanding leaders’, decision makers’ and evaluators’ patterns of behavior. Lab-in-the-field experiments facilitate insights into this area by allowing researchers to control information and action space (meaning that male and female decision-makers can be assessed based on similar actions), enabling causal inferences relating to the gender of the decision-maker to be drawn.

## Challenges of lab-in-the-field experiments

A number of ethical and practical challenges emerge when conducting lab experiments in the field. In this section, we reflect on our experience conducting the experiments reported in Sect. 4 to offer some insights into the challenges we faced and the strategies we adopted in response to them.

### Ethical concerns and ethics clearance

Primary data collection requires extensive safeguards to protect the human subjects participating in the research. This is especially true of experiments conducted in developing countries which present different, and potentially more serious, ethical issues than studies run in more developed countries, in part due to the majority of participants being poor, illiterate, unable to provide consent and having limited access to formal legal processes. While some of these concerns apply to any field-based economics research involving disadvantaged and vulnerable communities, others are specific to laboratory experiments conducted in the field.

A particular issue to consider is how ethical clearance is obtained. Where lab-in-the-field experiments are conducted in the country where the researchers are located, this is straightforward, as it is sufficient for researchers to approach the ethical review boards at their home institutions. However, lab experiments conducted internationally present additional challenges for review boards in developed countries (Krogstad et al., 2010). As a consequence, lab-in-the-field experiments conducted in a developing country may also require ethics clearance from that country. In many cases, co-authors on the project will be located and employed in the country where the experiment is scheduled, and their institution may insist on research oversight. This was the primary reason for also seeking ethics clearance from the Indian School of Business on our project. Local review boards may also have insights into research context and subject protection that external boards lack. However, local review boards may also be unfamiliar with the processes and standards involved in field experiments in economics, and may independently decide which consent process is required, or rely on standards from other disciplines (such as psychology or medicine) which unduly inconvenience or prevent the research. Navigating this process can be difficult and time consuming for researchers, and this should be kept in mind when planning projects.

*Ethics of payment*: Field researchers have grappled with the ethics of paying subjects for participating in research (Grady, 2019). However, the offer of payment may also influence subjects’ decision to participate in the research.^13^

In the field of experimental economics, financial payment for participation and decision-making is a well-established practice (Smith, 1976). Payments reimburse subjects for out-of-pocket expenses (such as travel to the experimental site), compensate them for their time and the burdens of participation, incentivize participation (Gelinas et al., 2018) and make choices in the experiment salient, mirroring decisions made outside the lab (Smith, 1976). In the case of lab experiments conducted in developing countries, the payments present several challenges. Foremost is what the level of payment should be. Few incentivized experiments are conducted in developing countries, so there are not many benchmarks to guide researchers.^14^ In our experiment, we aimed at average earnings of approximately Rs. 400 (USD 6 under current exchange rates) per participant for approximately 2–3 h of work. This corresponded to two days’ wages for unskilled workers in rural areas of Bihar.

Relatively high payments can lead to high turn up rates. In cases where more people want to participate in a session than are required, a transparent selection mechanism is necessary (similar to those used to decide which participants to turn away in the event of over-subscription to a lab experiment). For example, a public lottery could be used to determine participation.

In addition, even within countries, researchers need to vary earnings by local economic conditions (Charness et al., 2013). For instance, show-up fees to ensure participation among agricultural workers might be very different from those offered to office workers. This is because, if the researcher offered a uniform rate, it might, for example, influence the types of office workers who show up in a way that impacts the experimental findings (e.g. they might be unemployed or particularly enthusiastic about participating in research). Again, there are few benchmarks to guide such decisions. Our experiments were conducted in villages with similar economic conditions. All our sessions were conducted during the agricultural lean season when there is an excess supply of labour, ensuring that the opportunity cost of time and the effective payment rate were similar across locations.

*Sample selection and recruitment*: Sample selection is challenging for all primary data collection work in economics, but the hurdles are greater for experiments conducted in the field. Numerous researchers have highlighted the challenges of representative samples in university laboratory settings, and how these might be amplified in field experiments (see Cardenas and Carpenter (2008) and Harrison and List (2004)).

With respect to the first experiment we conducted in Bihar, our budget and time constraints meant that, at best, we could conduct 40 sessions with 960 participants in only a few locations. For a state with over 100 million inhabitants, this sample could not constitute a representative one. As a consequence, we could not fully claim sample representativeness. Instead, we focused on the internal validity of the experiment with more limited claims exploiting within experiment variation in treatment status. In addition, we used administrative census data to make informed statements about the representativeness of the sample. Given that our objective was to investigate men and women’s reactions to male and female leaders, having an equal number of men and women participate in the experiment was essential. While enrolling men was easy, targeting women was more challenging given the prevailing social norms and restrictions on women’s outside interactions and movements.^15^

To address this challenge, our recruitment strategy was to ensure that our research team included a number of female research assistants who could speak the local language and go door-to-door informing women of the opportunity to participate. We also distributed flyers that contained information about the eligibility criteria, the remuneration range and the time and location of the sessions. These flyers were also posted at prominent villages landmarks (e.g., post office, bus stops, schools, teashops, places of worship). Another possible strategy is to use reliable local agents (paid or unpaid) who can help select participants according to criteria determined by the researchers.^16^ We did not use agents as several of our research assistants were from Bihar.

*Adapting instructions*: Lab-in the-field experiments conducted across cultures can often involve languages other than English, and therefore require translation since most economics research is communicated in English. Roth et al. (1991) discuss several concerns associated with language translation that emerged while conducting experiments in Israel, Japan, Slovenia and the USA. As researchers conducting experiments in India, our protocols were developed in English, with final results also written in English. However, almost no participant was fluent in English, and therefore the experiments were conducted in the dialect of Hindi that was most prevalent in Bihar. We decided to recruit research assistants with spoken and written fluency in the local Hindi dialect as well as a background in economics so that they understood the research questions and respected the demands of the experimental methodology, but were also attuned to the local social and linguistic nuances. To prepare the translated materials, the research assistants suggested Hindi terms for technical economics ideas during our initial discussions. Then one team of research assistants translated the instructions and questionnaires into Hindi, while a second team translated the Hindi version back into English. Insofar as the original English version matched the re-translated version, we could be confident that the Hindi version was a faithful translation of the original. Three out of four of the researchers were also fluent in Hindi, and independently verified the translation.

While piloting the experiment, we discovered that despite being literate, many participants were not practiced readers and writers, and therefore pictorial instructions were critical.^17^ The instructions were read slowly and deliberately, and included several examples that showed how experimental choices translated into earnings. We included pauses where participants could ask clarifying questions. All experiments were conducted using paper and pencil because participants were familiar using these. Laptops were both unfamiliar to participants, and transporting, charging and storing them was a logistical challenge. One downside to paper-based experiments was that each experiment took longer to conduct than it would have with digitized data collection.

*Experimenters*: Field experiments require more research personnel, and people with different skills than those needed in lab settings. Reflecting this, Derry and Baum (1994) discuss the variety of situations experimenters in field settings may encounter, and how trained experimenters learn to anticipate and deal with these situations. Our experiments required us to cover 40 villages (with one day of canvasing and one day of experiments in each village) with appropriate periods for rest in one month. Given this time constraint, we created two teams, each of which included an experimenter and male and female research assistants for canvasing and conducting post-experiment surveys. The research assistants visited the experiment site the day before the experiment was scheduled to ensure that the venue of the session was satisfactory, and to recruit participants. The two teams operated in parallel to complete the experiments in time.

The research assistants were primarily recruited from Masters of economics programs in Patna, Bihar. While they were trained in microeconomics, they had little exposure to experimental economics. To develop their skills, the researchers held a 3-day training program with: (i) conceptual training on the value of the experimental method in economics; (ii) detailed training on the specifics of the experiment we planned to conduct, including mock experiments; and (iii) pilot testing conducted with recruited participants in a village outside Patna.

The safety of the research team and participants was paramount. The team carried expensive laptops, mobile phones and tablets, and a significant amount of cash to pay the experimental participants. For this reason, we started and finished early, so that the research team could return to their base before nightfall. In 2020, the spread of COVID-19 and the lockdowns imposed by governments added another layer of complexity. However, we adopted the principle of safety first, and (temporarily) discontinued field work.

## Conclusion

In this paper, we discuss the role played by lab-in-the-field experiments in economics, which the literature also refers to as artefactual, framed and extra-lab experiments. These methods have been used in many areas of economics and have provided researchers significant insights into social and economic phenomena, by focusing on outcomes, mediators (mechanisms) and moderators (heterogeneity). We present examples of how lab-in-the-field experiments can be conducted on their own or combined with conventional lab experiments, natural experiments, RCTs and surveys. We also highlight some of the unique opportunities and challenges presented by this approach, drawing on our recent research on gender and leadership as an example.

We anticipate two key avenues for growth in this area. In recent years, the use of online experiments and experiments embedded in household surveys has increased, and this is expected to continue. This allows researchers to investigate questions using a different lens and with a diverse sample. Another direction of growth may be in the increased use of technology, both for conducting experiments (using *apps*) and for recruiting participants (using GIS software).

## Footnotes

1 All experiments, including conventional laboratory experiments, involve random assignment of participants to one of the treatments. We follow the literature in economics, where RCTs are used to refer to experiments in the field where the intervention is done by researchers or policy makers, with participants who are familiar with the field context and requires them to be engaged to some degree (e.g., via providing survey responses).

2 There are several excellent review articles that focus on the use of field experiments in specific areas. Examples include Bertrand and Duflo (2016) on discrimination, List and Price (2016), Price (2014) and Brent et al. (2017) on environmental economics, and List and Rasul (2011) on labour economics.

3 Differences in behaviour across subject pools has been of considerable methodological interest to experimental economists (see for example, Ball and Cech (1996), Frechette (2011) and Snowberg and Yariv (2021)).

4 RCTs are usually designed to ensure balance amongst observable variables across treatment and control groups, and researchers use the baseline surveys to check for this balance. Lab-in-the-field experiments can help in opening the black box referred to as un-observable variables.

5 Falk and Heckman (2009), Friedman and Sunder (1994), Reuben and Winden (2008) and Xiao and Houser (2005) demonstrate this using lab experiments.

6 While many experiments have identified preferences using time-dated monetary payments, identifying time preferences from such monetary choices has also been argued to be problematic (Cubitt and Read (2007)). To address this, Augenblick et al. (2015) investigate choices over consumption (real effort), in addition to time-dated financial rewards, and find significant evidence of present bias in the domain of effort, but not in the domain of money. In particular, subjects allocate more work to the first work date, when the allocation of tasks is made in advance as compared to when it is made on the first work date itself. They also document a relationship between present bias and the demand for commitment as those who are potentially aware of their present bias, take actions to limit their future behaviour.

7 The literature using experiments (summarised in a recent survey by Eckel et al. (2021)) has shown that both preferences and beliefs play a critical role in explaining the gender gap in leadership.

8 Note that when a seat is reserved for women, only women can stand as candidates. However, all electors (male and female) are eligible to vote in the elections. Additionally, while quotas for women were introduced at the lower levels of governance, there were no corresponding quotas for women in state assembly and national parliament elections.

9 Although, strictly speaking, a female headed village should denote a village where the position of the head of the village council is *reserved* for women, we use the two meanings interchangeably because, while it is theoretically possible for a woman to be elected to the position of village head in the absence of a quota, this almost never happens in practice.

10 In both treatments, at every stage decision sheets are handed out and collected simultaneously from all group participants, helping preserve the anonymity of all participants. The leader’s decision and gender are only revealed to the members through the decision sheets. In the *Gender revealed* sessions, gender was (subtly) revealed using gender icons for the leader.

11 For more details see Gangadharan et al. (2016) and Gangadharan et al. (2019).

12 Participants had to rate the behavior of group members (both male and female) contributing 0 through to 200 in intervals of 50. Responses were elicited for both male and female led groups. Participants were also asked to rate the social appropriateness of a leader (both male and female) contributing a certain amount (Rs. 50/100/150) when they propose 100 and 200.

13 These challenges have been recognized in the context of biomedical research, raising “ethical concerns, such as undue inducement, exploitation, and biased enrollment” (Resnick (2015)).

14 A useful resource on this issue is Cardenas and Carpenter (2008).

15 Recruiting participants who are unable to provide consent (e.g., children) can present additional challenges.

16 For example, Cameron et al. (2013) employed a survey company that used an advertisement designed by the researchers to recruit participants through their regular subject-recruitment-network. The company also approached people on the street, and posted the advertisement on their website, notice boards, and street lampposts across the city. Similarly, Chaudhuri et al*.* (2020) and Chaudhuri et al. (2021) use a survey company that contacted the council officials and identified elected local politicians to find the appropriate sample for their research. Gangadharan et al*.* (2020) obtained survey contact lists from the local government statistical organization. They also hired local research assistants who received help from teachers, principals and village elders who were familiar with the demographic distribution of their communities.

17 Cardenas and Carpenter (2008) raise issues of language sophistication and the cognitive ability of participants, while also arguing in favour of pen-and-paper experiments that are easier to administer.
